# Comparison of lateral entry and crossed entry pinning for pediatric supracondylar humerus fractures: a meta-analysis of randomized controlled trials

**DOI:** 10.1186/s13018-022-03286-z

**Published:** 2022-08-19

**Authors:** Andreas Rehm, Elizabeth Ashby, Luke Granger, Joshua C. Y. Ong

**Affiliations:** Department of Paediatric Orthopaedics, Cambridge University Hospitals NHS Trust, Cambridge, UK

We read with interest the recent publication by Zhao et al. [1]. Zhao et al. [1] included 933 children, but 52 are duplicated since the papers by Yen and Kocher [2] and Kocher et al. [3] have reported the same patient cohort. The study by Dučić et al. [4], with 138 patients, compared two crossed K-wire techniques and not crossed K-wires with diverging lateral K-wires as all other included studies. Therefore, the 2 former studies should have been excluded, limiting the meta-analysis to 10 studies with 743 patients.

Zhao et al. [1] quoted Skaggs et al. [5] as having defined displacement of Baumann’s angle (BA) as no displacement (< 6°), mild displacement (6° to 12°) and major displacement (> 12°). However, Skaggs et al. [5] did not define such grading but defined instead a “meaningful change” as a difference of ≥ 12° between the perioperative and final BA. It was Kocher et al. [3] who defined no-, mild- and major displacement as above, stating that the grading was according to the criteria reported by Skaggs et al. [5]. Skaggs et al. [5] made the mistake to base their definition of the ≥ 12° cutoff on a wrong angle presented in Camp et al.’s abstract [6], where an important transcription error had occurred, which was neither recognized by Camp et al. [6], nor Skaggs et al. [5] or Kocher et al. [3], invalidating the latter authors’ definition of “meaningful change” and the definition of no-, mild- and major displacement which were used by Zhao et al. [1] to judge the outcomes between the crossed and lateral K-wire groups.

Camp et al. [6] measured that the perceived BA increases with internal and decreases with external humeral rotation if an antero-posterior radiograph (APR) is not taken as a true but rotated APR, with the perceived BA changing by ~  ± 1.6° per 10° change of rotation with the humerus parallel to the collector/X-ray cassette and by ~  ± 5° per 10° change of rotation with the humerus flexed 30° in relation to the collector/X-ray cassette. The 1.6° BA change for every 10° change of humeral rotation was erroneously transcribed by Camp et al. [5] into their abstract as 6°, with the 6° then having formed the basis for Skaggs et al.’s [5] definition of “meaningful change.” Using Skaggs et al.’s [5] model and the correct angle of 1.6° would re-define “meaningful change” as > 3° instead of ≥ 12°, indicating the possibility that clinically significant group differences were dismissed as irrelevant because of Skaggs et al.’s erroneous modeling. Skaggs et al. [5] did also not consider that the angle decreases or increases depending on the direction of the rotation away from a true APR. Camp et al. [6] stressed the importance of taking consecutive APRs in matched positions and highlighted that not appreciating rotatory malalignment might make the fracture, e.g., appear to be in more varus than it actually is or a true varus deformity might appear to be normal. Zhao et al.’s [1] reliance on Skaggs et al.’s [5] definition to assess BA will have resulted in underreporting of displacement and deformity and therefore invalidates conclusions based on BA displacement, since possible significant group differences might not have been identified.

Zhao et al. [1] did not investigate rotational displacement, with none of the included papers having considered to measure the lateral rotation percentage [7].

Zhao et al. [1] graded functional outcome according to the criteria of Flynn et al. [8]. This requires accurate and reliable measurement of the angles and for the authors to know the normal range of elbow movements in children. None of the studies included by Zhao et al. [1] assessed intra- and interobserver correlation coefficients for their measurements of elbow movements and only 3 provided data for the total range of movements (ROM), with the available data raising substantial doubts about the accuracy and reliability of these measurements and if surgeons were familiar with the normal ROM. McKay et al. [9] measured a mean elbow flexion of 146° and mean extension of 3° for normal children 3–9 years of age (total ROM of 149°). Tripuraneni et al. [10] reported a final mean ROM of 116.5°/117° at a mean follow-up of 54.6/65.1 days for crossed and lateral pin fixation, respectively. Maity et al. [11] reported a total mean ROM of 130° and 129° for the crossed and lateral K-wire group, respectively, at 3 months, documenting excellent (80%/73%), good (9%/12%) and fair (11%/15%) Flynn grading. Kocher et al. [3] reported a mean total ROM of 124°/129° for the crossed/lateral K-wire group at 3 months, with 79%/82% excellent, 17%/14% good and 4%/4% fair Flynn grading. The documented angles are inconsistent with the given Flynn grades, since a total ROM of 130° should have been graded as poor, based on the normal total ROM of 149° [9]. This indicates that the authors [3, 9, 10] were not familiar with the normal values for elbow ROM and that ROM was most likely estimated and not measured, which is supported by only one [4] of the papers included by Zhao et al. [1] having reported the use of a goniometer to measure the carrying angle but not ROM. Estimating the full ROM of an uninjured elbow as 130° would give a measuring error of 19°.

In conclusion, considering that Zhao et at [1] reported that the quality of the included studies was generally poor, the fact that Skaggs et al. [4] and Kocher et al. [3] introduced fundamentally flawed definitions for a change of BA based on a major data error and authors having either presented no or inaccurate estimates of ROM to judge outcome of elbow function, we are of the opinion that the data available to Zhao et al. [1] and the use of these data for a meta-analysis are unreliable to compare outcomes for crossed and diverging lateral entry K-wire fixation, apart for nerve injuries.

## Data Availability

Not applicable.

## Abbreviations

APR: Antero-posterior radiograph
BA: Baumann’s angle
ROM: Range of movement
